# Effects of the continuous subcutaneous infusion of foslevodopa-foscarbidopa on swallowing in patients with Parkinson’s disease

**DOI:** 10.1016/j.prdoa.2025.100378

**Published:** 2025-07-29

**Authors:** Makito Hirano, Makoto Samukawa, Chiharu Isono, Rino Inada, Yuta Fukumoto, Keisuke Yoshikawa, Hitoshi Namura, Hanami Sakata, Takahiro Hisatomi, Toru Michiura, Hiroto Nakamura, Akira Morita, Genki Hoshino, Kensuke Yamana, Atsushi Terayama, Yuji Higashimoto, Yoshiyuki Mitsui, Yoshitaka Nagai

**Affiliations:** a Department of Neurology, Kindai University, Faculty of Medicine, Japan; b Division of Rehabilitation Medicine, Kindai University Hospital, Japan; c Department of Rehabilitation Medicine, Kindai University, Faculty of Medicine, Japan

**Keywords:** Dysphagia, Parkinson’s disease, DOSS, Deglutition, CSCI of foslevodopa/foscarbidopa

## Abstract

**Background:**

Dysphagia is a potentially fatal symptom of Parkinson’s disease (PD) and is characterized by frequent silent aspiration, a known risk factor for aspiration pneumonia. A previous study has reported that the dopamine agonist rotigotine (levodopa equivalent dose of 60 mg/day) delivered via transdermal patch improves swallowing function more effectively than oral levodopa (200 mg/day), highlighting the importance of continuous dopaminergic stimulation (CDS) in managing dysphagia. To achieve CDS, patients with advanced PD may require device-assisted therapies (DATs), including levodopa–carbidopa intestinal gel (LCIG), which have significantly improved swallowing function on some measures. In contrast, swallowing function is an important prognostic factor for patients with PD undergoing LCIG. Continuous subcutaneous infusion of foslevodopa/foscarbidopa (CSCI-FF) is a newly developed DAT; however, its effect on swallowing function remains unknown.

**Methods:**

This retrospective open-label evaluator-blinded study included seven patients with PD. Swallowing function was assessed using videofluoroscopic swallow studies (VFSS) conducted before and after initiating CSCI-FF. Evaluations included the Japanese Swallowing Scale, the Dysphagia Outcome and Severity Scale (DOSS), Penetration/Aspiration Scale, oral transit time (OTT), and pharyngeal transit time (PTT).

**Results:**

Following the introduction of CSCI-FF, results of VFSS showed significant improvement in the total score of the Japanese swallowing scale, OTT, and PTT.

**Conclusions:**

This study provides preliminary evidence that CSCI-FF may partially improve swallowing function in patients with advanced PD. Further research with larger cohorts is warranted.

## 1 Introduction

Parkinson’s disease (PD) impairs swallowing function through disruptions in both voluntary and involuntary movements and altered sensation in the pharyngeal and laryngeal regions [1,2]. Abnormal swallowing, or dysphagia, is a potentially fatal symptom of PD that is characterized by frequent silent aspiration, leading to aspiration pneumonia [3]. Several studies have shown that dopamine agonists such as apomorphine and rotigotine can alleviate dysphagia in patients with PD [1,[4], [5], [6]]. These agents improve voluntary oral functions and involuntary pharyngeal functions, as evaluated by videofluoroscopic swallow studies (VFSS) [4]. Notably, 2 mg/day of transdermal rotigotine (levodopa equivalent dose [LED] 60 mg/day) has been reported to be more effective than 200 mg/day of oral levodopa (with carbidopa) in improving dysphagia, underscoring the potential benefits of continuous dopaminergic stimulation (CDS) in managing dysphagia [5]. Consistent with this finding, monoamine oxidase B (MAOB) inhibitors such as rasagiline and safinamide facilitate CDS and have been shown beneficial effects on swallowing function [[7], [8], [9]]. However, such findings were derived from patients with relatively mild or early-stage PD.

In advanced stages of PD, achieving CDS often requires device-assisted therapies (DATs), such as the levodopa–carbidopa intestinal gel (LCIG). Although the impact of LCIG on swallowing function has been explored in limited studies, only one report using flexible endoscopic evaluation of swallowing demonstrated significant improvement in select measures among patients with PD [10]. In contrast, the presence of dysphagia in patients receiving LCIG has been linked to poor long-term outcomes [11]. Recently, a new DAT—continuous subcutaneous infusion of foslevodopa/foscarbidopa (CSCI-FF)—has been introduced in Japan. When administered subcutaneously, foslevodopa and foscarbidopa are converted into levodopa and carbidopa, which provide dopaminergic therapy for PD. However, the effects of CSCI-FF on swallowing function have yet to be investigated.

In this open-label evaluator-blinded study, we examined the effects of CSCI-FF on swallowing function in seven patients with PD using VFSS.

## 2 Patients and methods

### 2.1 Patients

Table 1 summarizes the clinical characteristics of the patients. This retrospective, observational, open-label study enrolled seven consecutive patients with PD (four men and three women; mean age, 67 ± 7 years). All patients underwent VFSS both before and after initiating CSCI-FF. CSCI-FF was initiated to replace oral levodopa and catechol-O-methyltransferase (COMT) inhibitors, while other antiparkinsonian medications were continued as needed (Table 1). To compare swallowing function between oral medications and CSCI-FF, VFSS was performed before and during the introduction of CSCI-FF. Optimization of CSCI-FF was performed after the post-treatment VFSS. Levodopa equivalent doses (LED) were calculated based on established conversion factors [12], with a conversion factor of 170 mg/mL/h applied for CSCI-FF. All patients were diagnosed with PD by board certified neurologists in accordance with the UK Parkinson’s Disease Society Brain Bank Clinical Diagnostic Criteria. Hoehn and Yahr stages ranged from II to IV, and all patients experienced motor fluctuations. None exhibited extreme autonomic dysfunction, upper motor neuron signs, dementia, or comorbid painful or disabling conditions. Patients with diabetes mellitus or a history of stroke were enrolled only when these concomitant diseases were stable and treatment regimens were not changed. At baseline, all patients were receiving oral levodopa therapy (Table 1).

**Table 1:** Clinical information of patients with Parkinson’s disease treated with CSCI-FF.

Patient#	1	2	3	4	5	6	7
Sex	M	F	F	F	M	M	M
Age at examination	60	67	64	65	71	60	79
Age at onset (y)	49	50	58	59	59	52	68
Duration of disease (y)	11	17	6	6	12	8	11
Duration of CSCI −FF(d)	7	6	2	36	8	6	4
Hoen-Yahr grade (ON) pre/post	4/3	3/4*	3/3	4/3	2/3*	2/nd	3/3
UPDRS III (ON) pre/post	65/45	23/39*	6/5	50/42	20/11	37/nd	27/15
UPDRS III (Speech) pre/post	2/1	0/1*	0/0	0/0	1/0	2/nd	1/1
Oral phase score pre/post	7/8	7/7	8/8	6/7	6/9	7/8	7/7
Pharyngeal phase score pre/post	10/9*	8/9	9/10	9/11	8/8	9/9	8/9
Total score pre/post	17/17	15/16	17/18	15/18	14/17	16/17	15/16
DOSS pre/post	5/4*	4/5	5/5	5/5	3/4	5/5	5/3*
PAS pre/post	1/5*	8/3	2/1	2/2	8/8	1/1	2/4*
OTT (s) pre/post	0.401/ 0.366	0.399/ 0.368	0.668/ 0.500	1.067/ 0.904	1.602/ 0.801	1.501/ 0.801	0.430/ 0.400
PTT (s) pre/post	0.800/ 0.668	0.735/ 0.734	0.634/ 0.600	0.567/ 0.500	0.570/ 0.570	0.634/ 0.567	0.830/ 0.770
LED (mg) pre/post	810/ 1149.5	985/ 1030.1	500/ 762	800/ 1040.4	1032/ 1145.5	1031.5/ 1451.5	882/ 1079.6
LDD (mg) pre/post	400/ 739.5	665/ 870.1	350/ 612	800/ 1040.4	932/ 1045.5	998.5/ 1311.5	632/ 879.6
Medication Pre-CSCI-FF (except for levodopa with/without COMT inhibitors, mg)	Sf 50, RP 32, Z 50	R 16	Rs 0.5, Am 100	−	Z 50	RP 24, P 0.125	Sf 100, Z 50
Medication Post-CSCI-FF (except for CSCI-FF, mg)	Sf 50, RP 32, Z 50	R 8	Rs 0.5, Am 100	−	Z 50	RP 8, Rs 1	Sf 100, Z 50
Note		Dis				Dis	
LED, levodopa equivalent dose; LDD, levodopa daily dose including only levodopa-containing preparations (including carbidopa/levodopa/COMT inhibitor); CSCI-FF, continuous subcutaneous infusion of foslevodopa/foscarbidopa; pre/post, before/after the introduction of CSCI-FF introduction; *, worsening of the score; Dis, discontinuation; UPDRS-III, part III of United PD Rating Scale; Sf, safinamide; RP, ropinirole patch; Z, zonisamide; R, oral ropinirole extended release; Rs, rasagiline; nd, not done; COMT, Catechol-O-methyltransferase.							

### 2.2 Ethics

This observational study was approved by the Institutional Review Board of Kindai University. All participants provided written informed consent for publication of their clinical information.

### 2.3 VFSS

All patients underwent VFSS both before and after the initiation of CSCI-FF, conducted during their ON periods. Each VFSS was independently evaluated by a speech-language pathologist and a neurologist, both of whom were blinded to clinical details and had over 10 years of experience in swallowing assessment. In cases of scoring discrepancies, a third evaluator—a neurologist with expertise in swallowing disorders—made the final judgment. Intra- and inter-rater reliability among the three evaluators showed strong correlations, confirming consistent scoring practices [8]. VFSS was conducted following previously established protocols [8,13,14]. In brief, patients first swallowed 5 mL of a diluted barium solution three times. If no severe swallowing impairment was observed, they proceeded to swallow a more concentrated barium solution three times, without volume restrictions, simulating typical swallowing behavior. The worst score observed for each parameter was recorded. Additional assessments included swallowing 5 mL of barium mixed with a commercial xanthan gum-based thickener (IDDSI Level 1 [15]) and 3 g of barium jelly. Swallowing function was evaluated using both a Japanese scale developed by the Japanese Society of Dysphagia Rehabilitation and the internationally recognized Dysphagia Outcome and Severity Scale (DOSS) [9,14,16]. The Japanese scale assesses the following: lip closure, bolus formation, bolus transport during the oral phase, constriction of the pharynx, elevation of the larynx, bolus stasis at the valleculae and pyriform sinus, and aspiration during the pharyngeal phase. A three-point scale was used to semiquantify each variable in a VFSS series: 3 (normal), 2 (disturbed), and 1 (severely disturbed). When the Japanese scale was used, the oral phase (3 = severely affected and 9 = normal) and pharyngeal phase (4 = severely affected and 12 = normal) were separately evaluated, and the values summed to derive the total score [5]. The DOSS, which ranges from 1 (severely impaired) to 7 (normal), has been used internationally but does not distinguish between the oral and pharyngeal phases [14]. The Penetration–Aspiration Scale (PAS) was also used to assess airway compromise, with scores ranging from 1 (normal) to 8 (silent aspiration) [17].

Oral transit time (OTT) was defined as the interval from the initiation of backward tongue movement to the arrival of the bolus head at the ramus of the mandible [4]. Pharyngeal transit time (PTT) was measured from the moment the bolus head reached the ramus of the mandible to the point at which the tail of the bolus passed through the upper esophageal sphincter, as previously described [4]. For each patient, OTT and PTT were assessed during the second swallow of 5 mL of diluted barium. Reference values for normal OTT and PTT were obtained from a prior study involving six healthy individuals (mean age: 65 ± 14 years) [7].

Comparisons of all swallowing measures before and after the initiation of CSCI-FF were conducted using the Wilcoxon signed-rank test, due to the ordinal or non-normally distributed nature of these variables as reported in previous studies [5,6].

### 2.4 Evaluation of parkinsonism

Parkinsonian symptoms were assessed using the Hoehn–Yahr scale and Part III (Motor Examination) of the Unified Parkinson’s Disease Rating Scale (UPDRS-III), based on medical chart documentation. All patients, except one, were evaluated both before and between 2 and 36 days after the initiation of CSCI-FF.

## 3 Results

### 3.1 Improvement in swallowing function

**Fig. 1:**
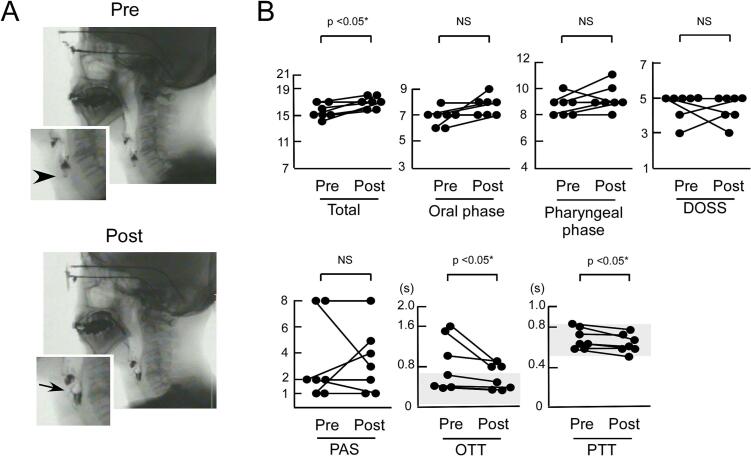
Swallowing function in seven patients with PD before (pre) and after (post) the initiation of continuous subcutaneous injection of foslevodopa/foscarbidopa (CSCI-FF). (A) Representative images of videofluoroscopic study of swallowing (VFSS) for Patient 2. Aspiration was observed in pretreatment (arrowhead), whereas it disappeared in posttreatment; however, laryngeal penetration remained (arrow). (B) Results of VFSS showed significant improvement (*p* < 0.05, Wilcoxon signed-rank test) in total score, oral transit time (OTT), and pharyngeal transit time (PTT). The oral phase score, pharyngeal phase score, Dysphagia Outcome and Severity Scale (DOSS) score, and Penetration/Aspiration Scale (PAS) scores did not significantly change. Increases in total score, oral phase score, pharyngeal phase score, and DOSS (upper panels) and decreases in PAS score, OTT, and PTT indicate improvement (lower panels). Shaded areas are normal ranges in OTT and PTT according to a previous study (Parkinsonism Relat Disord 78 [2020] 98).

Following the initiation of CSCI-FF, the total score on the Japanese swallowing scale showed a significant improvement (*p* < 0.05, Wilcoxon signed-rank test) (Fig. 1). However, no significant changes were observed in the oral phase score, pharyngeal phase score, PAS, or DOSS scores. OTT and PTT significantly improved after the introduction of CSCI-FF (*p* < 0.05, Wilcoxon signed-rank test) (Fig. 1 and Table 1). Despite these improvements, two patients discontinued CSCI-FF after VFSS evaluation. Patient 3 showed no motor improvement based on UPDRS-III scores, and Patient 6 reported no subjective benefit from the treatment. In Japan, there are only two sizes of cannulas, 6 mm and 9 mm in length. For the two patients who discontinued CSCI-FF, a 9 mm long cannula was used and was not resized. The injection site was rotated at least 5 cm away from the umbilicus according to the pharmaceutical company’s instructions, with no benefit for the two patients.

### 3.2 Changes in motor function as evaluated by the Hoehn–Yahr scale and UPDRS-III

Motor function, as measured by the UPDRS-III, showed a trend toward improvement following the introduction of CSCI-FF (32 ± 22 *vs.* 26 ± 18), although this change did not reach statistical significance (Wilcoxon signed-rank test). The Hoehn–Yahr scale scores remained unchanged (3.2 ± 0.75 *vs.* 3.2 ± 0.75, Wilcoxon signed-rank test). Because speech and swallowing function has been correlated, UPDRS-III speech scores were evaluated. The score of speech improved in two of the six patients, remained unchanged in three, and worsened in one who discontinued CSCI-FF. (Table 1). Interestingly, the degree of improvement in UPDRS-III scores (N = 6) was significantly correlated with a worsening in PAS scores (*p* < 0.05, Spearman’s rank correlation coefficient). However, no significant associations were observed between changes in UPDRS-III scores and changes in VFSS total scores, OTT, PTT, or DOSS scores (Spearman’s rank correlation coefficient).

## 4 Discussion

This retrospective, open-label, evaluator-blinded study involving seven patients with PD demonstrated that CSCI-FF led to partial but significant improvements in swallowing function, as assessed by VFSS. Notable improvements were observed in the total score of the Japanese swallowing scale, as well as in OTT and PTT. Reported research has shown that PD medications primarily improve the oral phase of swallowing, which relies largely on voluntary movement [18]. In this study, although the oral phase score on the Japanese scale did not significantly improve, OTT showed a significant reduction, suggesting that OTT, as a continuous and quantitative measure, is more sensitive to subtle changes than the composite oral phase score, which encompasses more complex, multidimensional components. In several studies, levodopa also improves swallowing function during the pharyngeal phase [5,19], which is sequential reflexes of striatal muscles in the neck, consistent with the observed improvement in PTT. The lack of significant change in the pharyngeal phase score, despite improved PTT, may reflect the same discrepancy seen between the oral phase score and OTT. These findings support the notion that central dopaminergic stimulation facilitates both oral and pharyngeal phases, thereby contributing to improved swallowing function.

**Fig. 2:**
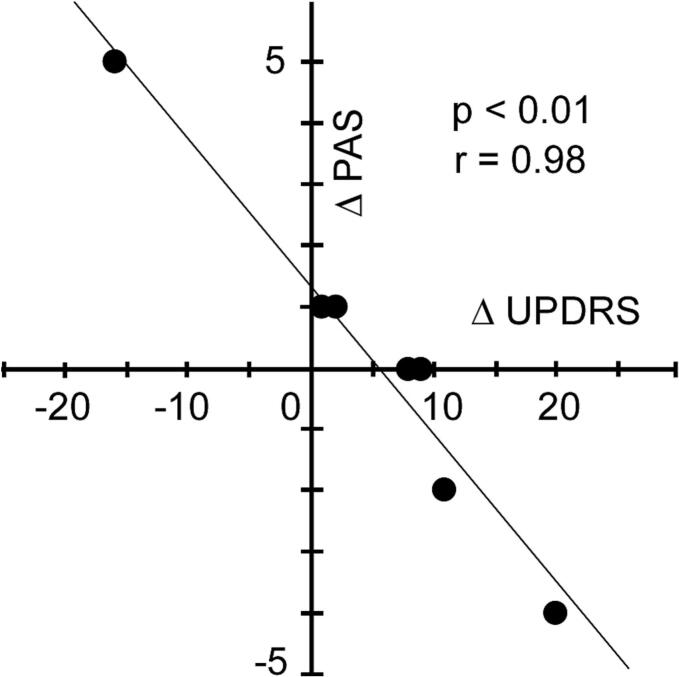
Correlation between changes in UPDRS Part III scores and Penetration/Aspiration Scale (PAS) scores before (pre) and after (post) the initiation of continuous subcutaneous infusion of foslevodopa/foscarbidopa (CSCI-FF). Among six patients (N = 6), greater improvement in UPDRS Part III motor scores was significantly associated with a worsening in PAS scores (*p* < 0.05, Spearman’s rank correlation coefficient).

Notably, neither the PAS nor DOSS scores showed significant improvement across all patients, likely due to mild worsening of laryngeal penetration in two patients—despite observed improvements in OTT. This suggests that an improved oral phase, characterized by overly rapid oral transit of the bolus, may compromise the airway if not accompanied by sufficient improvement in laryngeal closure during the pharyngeal phase. Because OTT is partially governed by voluntary movements, we speculate that voluntary motor improvement not matched by corresponding improvement in the pharyngeal phase promotes laryngeal penetration. Consistent with this, changes in PAS scores were inversely correlated with changes in UPDRS-III motor scores (Fig. 2), indicating that greater motor improvement might be associated with an increased risk of laryngeal penetration. This highlights the need for careful monitoring of swallowing safety in patients who exhibit marked motor improvement.

In this study, two patients discontinued CSCI-FF due to a lack of perceived or measured improvement in motor symptoms. Interestingly, both patients demonstrated improvements in swallowing function, reinforcing reported observations [1,5,7] that improvement in overall motor function and that in swallowing do not necessarily occur in parallel.

It could be assumed that switching from oral levodopa to CSCI-FF, with an associated increase in levodopa dosage, would naturally improve swallowing function. However, many of our patients were unable to tolerate higher doses of oral levodopa due to motor complications—some of which may be mitigated through CSCI-FF. Therefore, the ability to improve or maintain swallowing function using CSCI-FF represents a valuable therapeutic option in the management of advanced PD.

In a previous study, the rotigotine transdermal patch appeared to produce more consistent improvements in swallowing function than CSCI-FF did in our current cohort [5]. However, notable differences in patient characteristics may account for this discrepancy. For instance, the patients in that study had a shorter disease duration (4 years *vs.* 10 years) and lower disease severity, as reflected by UPDRS-III scores (21 *vs.* 39). These factors likely influenced the treatment response. Our findings suggest that dopaminergic therapy can still yield measurable benefits in swallowing function, even in advanced stages of PD. Thus, patients with milder disease phenotypes might respond more favorably to CSCI-FF in terms of swallowing function.

## 5 Conclusions

To the best of our knowledge, this is the first study to show that CSCI-FF can lead to partial but significant improvements in swallowing function, as evaluated by VFSS, in patients with advanced PD. However, the study is limited by its small sample size and the short duration of follow-up after initiating CSCI-FF. Further research involving larger patient cohorts and longer observation periods is necessary to better understand the long-term effects of CSCI-FF on dysphagia in this population.

## CRediT authorship contribution statement

**Makito Hirano:** Writing – original draft, Methodology, Investigation, Formal analysis, Data curation, Conceptualization. **Makoto Samukawa:** Writing – review & editing, Formal analysis, Data curation. **Chiharu Isono:** Writing – review & editing, Formal analysis, Data curation. **Rino Inada:** Writing – review & editing, Data curation. **Yuta Fukumoto:** Data curation. **Keisuke Yoshikawa:** Data curation. **Hitoshi Namura:** Data curation. **Hanami Sakata:** Data curation. **Takahiro Hisatomi:** Data curation. **Toru Michiura:** Data curation. **Hiroto Nakamura:** Data curation. **Akira Morita:** Data curation. **Genki Hoshino:** Data curation. **Kensuke Yamana:** Data curation. **Atsushi Terayama:** Data curation. **Yuji Higashimoto:** Writing – review & editing, Validation, Supervision. **Yoshiyuki Mitsui:** Supervision, Data curation. **Yoshitaka Nagai:** Writing – review & editing, Conceptualization.

## Funding

No funding was received for conducting this study.

## Declaration of competing interest

The authors declare the following financial interests/personal relationships which may be considered as potential competing interests: M. Hirano has received funding for speaker honoraria from Sumitomo, Ono, Otsuka, Novartis, Kyowa-Kirin, Eisai, and Takeda; and has received Grants-in-Aid from Japan’s Ministry of Education, Culture, Sports, Science and Technology; AMED in Japan; and research support from Kindai University. M. Samukawa has received funding for speaker honoraria from Takeda, FP, Eisai, and Sumitomo Pharmaceutical. C. Isono reports no disclosures. Y. Nagai has received funding for speaker honoraria from Sumitomo, Kyowa-Kirin, Tanabe-Mitsubishi, Amgen, and Takeda; and has received Grants-in-Aid from Japan’s Ministry of Education, Culture, Sports, Science and Technology; AMED in Japan. He also has received scholarship donation from Otsuka, Kyowa-Kirin, Tanabe-Mitsubishi, Fujimoto, Takeda, Sumitomo, Daiichi-Sankyo, Esai, and Chugai.
